# The effects of qishen granules for patients with chronic heart failure: A multicenter randomized double-blind placebo-controlled trial

**DOI:** 10.3389/fphar.2022.1017734

**Published:** 2022-12-23

**Authors:** Kangjia Du, Junjie Liu, Nannan Tan, Xinyi Huang, Juan Wang, Huihui Zhao, Wei Wang

**Affiliations:** ^1^ Department of Chinese medicine, Beijing University of Chinese Medicine, Beijing, China; ^2^ Department of Cardiology, Nanjing Pukou Hospital of Traditional Chinese Medicine, Nanjing, China

**Keywords:** chronic heart failure, qi shen granules, randomized controlled trial, detoxification, NT-probnp

## Abstract

**Background:** Despite advancements in chronic heart failure (CHF) treatment, the effect often remains unsatisfactory and unstable. More effective therapies are needed. Qishen granules (QSG) are a novel Chinese botanical drug effective in treating CHF in animal models, but clinical evidence remains inadequate.

**Objective:** This study aims to evaluate the effects of QSG on patients with CHF.

**Methods:** We enrolled CHF patients in this 12-week, randomized, double-blind, placebo-controlled trial and randomly assigned them to the QSG (twice a day, 6.8 g granules at once) or placebo group. The primary endpoint was a change in the plasma N-terminal pro-B-type natriuretic peptide (NT-proBNP) level after treatment. The secondary outcome consists of the New York Heart Association (NYHA) functional classification, 6-min walking distance (6MWD), TCM syndrome integral scale, quality of life, and echocardiographic index.

**Results:** A total of 191 patients completed the 12-week follow-up period, with 94 in the QSG group and 97 in the placebo group. The Qishen granules group demonstrated a considerably greater reduction in NT-proBNP than the placebo group (50% vs 32% for QSG vs placebo, respectively; *p* = 0.011). Patients who received QSG performed better in the NYHA functional rank, 6MWD, TCM syndrome integral scale, and quality of life (*p* < 0.05). The QSG group performed better in HFrEF patients regarding the efficiency of NT-proBNP. There was no statistical significance in the change in evaluated safety parameters, such as blood routine and biochemistry.

**Conclusion:** Based on standard treatment, Qishen granules further reduced the levels of NT-proBNP when compared with placebo. Together with other outcomes, our findings suggest that QSG could be used in combination therapy for CHF.

**Clinical Trial Registration**: www.clinicaltrials.gov, identifier NCT03027375. Registered 9 October 2017

## Introduction

Chronic heart failure (CHF), the final stage of various myocardial diseases, is a comprehensive clinical syndrome that occurs when the heart chamber cannot pump enough blood for organs (Groenewegen et al., 2020). According to recent epidemiological data, the prevalence of heart failure in China is 3.5% (Guo et al., 2016). Despite advances in the standard treatment strategy for heart failure, the effect often remains unsatisfactory and unstable. An international prospective cohort study data prove that 16.5% of people die of CHF within a year (Dokainish et al., 2017). Traditional Chinese medicine (TCM) has been widely used to treat CHF in China for years due to mechanisms of multitarget effects and a significant curative effect in alleviating symptoms (Li et al., 2013). From the general perspective of TCM, the primary cause of heart failure is heart Qi deficiency and blood stasis, which refers to a pathological state in which Qi cannot promote blood circulation, resulting in poor or even stagnant blood circulation. The current treatment has successfully improved symptoms and quality of life but has performed poorly in delaying ventricular remodeling and improving long-term prognosis. Researchers increasingly recognize that other unnoticed pathologies may play an essential role in the progression of heart failure. Heat toxicity is unnoticed pathogenesis closely related to inflammation. Heat toxicity is thought to induce acute episodes of heart failure, exacerbate symptoms and promote ventricular remodeling. It has been gradually considered an essential factor in heart failure (Yuan et al., 2014; Wang et al., 2021). Therefore, heat-clearing and detoxifying might be an effective therapy to complement the classical treatment of tonifying Qi and removing blood stasis. Qi Shen granules (QSG) are the representative drug for this innovative therapy.

QSG evolved from two well-known prescriptions (Simiaoyongan Decoction and Zhenwu Decoction). Researchers conducted experiments in animal and cell models to validate the effects of QSG and explore its mechanism of action. These studies indicated that QSG could treat CHF by facilitating cardiac contractile function, improving hemodynamics, and attenuating remodeling (Yang et al., 2020; Zhang et al., 2020; Chen et al., 2021). However, no clinical studies have been reported. Therefore, we designed a randomized control study to assess the efficacy and safety of QSG.

## Methods

### Design

This was a 12-week, randomized, double-blind, placebo-controlled, multicenter study. This study aimed to determine whether QSG reduces NT-proBNP, improves cardiac function and exercise performance, and alleviates clinical symptoms in heart failure patients. The participants could quit at any time for any reason. The appropriate ethics committees approved all studies. This study has been registered at Clinical Trials. gov (NCT: 03027375). More detailed design information was previously reported in 2017 (Wang et al., 2017).

### Drug composition

QSG consists of six TCM botanical drugs: Astragalus mongholicus Bunge [*Leguminosae*; *Astragali Radix*], Salvia miltiorrhiza Bunge [*Lamiaceae; Salviae miltiorrhizae radix et rhizoma*], Aconitum carmichaeli Debeaux [*Ranunculaceae*; *Aconiti Lateralis Radix Praeparata*], *Scrophularia ningpoensis Hemsl* [*Scrophulariaceae*; *Scrophulariae Radix*], *Lonicera japonica* Thunb *[Caprifoliaceae; Lonicerae Japonicae Flos*], Glycyrrhiza glabra L [*Leguminosae; Glycyrrhizae Radix et Rhizoma*]. The quantity of each drug is 6 g, 1.5 g, 0.9 g, 2 g, 2 g, and 1.2 g in sequence.

All drugs were extracted by water extraction and alcohol sedimentation. Water extraction process conditions: add eight times the amount of water, and extract three times, each time for 1 h. Alcoholic sedimentation process: concentrate the aqueous extract to a relative density of 1.10∼1.15 (60°C), add ethanol until its concentration is 70%, let it stand and refrigerate for 16 h, centrifuge, separate, take the supernatant, concentrate under reduced pressure and dry under vacuum (60°C, −0.8 MPa). The excipient is lactose.

### Population

CHF patients from three hospitals (China-Japan Friendship Hospital, Hubei Provincial Hospital of Traditional Chinese Medicine, and The First Affiliated Hospital of Beijing University of Traditional Chinese Medicine) were screened following eligibility and exclusion criteria. The eligibility criteria were as follows (Groenewegen et al., 2020): age between 18 and 75 years (Guo et al., 2016), clinically diagnosed with heart failure for more than 3 months (Dokainish et al., 2017), CHF caused by coronary heart disease and hypertension, which was diagnosed according to the Chinese guidelines published in 2014 (Li et al., 2013), New York Heart Association (NYHA) functional class from II to IV (Wang et al., 2021), a serum N-terminal pro-B type natriuretic peptide (NT-proBNP) level ≥450 pg/ml (Yuan et al., 2014), provision of written informed consent (Chen et al., 2021), inclusion of all types of ejection fraction. The exclusion criteria were as follows (Groenewegen et al., 2020): CHF accompanied by severe valvular heart disease, congenital heart disease, pericardial disease, cardiomyopathy, unstable angina, acute myocardial infarction (within the previous 4 weeks), cardiogenic shock, acute myocarditis, infective endocarditis, or uncontrolled severe cardiac arrhythmia with hemodynamic changes (Guo et al., 2016); pulmonary heart disease, pulmonary hypertension caused by acute or chronic pulmonary embolism or cerebral apoplexy in the last 6 months (Dokainish et al., 2017); severe hepatic inadequacy with alanine aminotransferase or alkaline phosphatase levels more than twice the upper normal limit, renal inadequacy with a serum creatinine level >3 mg/dl (>265 μmol/L), severe electrolyte imbalance, severe hematologic disease, malignant tumor, diabetes mellitus with severe complications, or severe endocrine diseases such as hyperthyroidism and hypothyroidism (Li et al., 2013); acute infection confirmed by any one of the following three indicators: a) fever, b) a white blood cell count>10 × 10^9^/L and a percentage of neutrophils> 75%, c) shadow on chest X-ray (Wang et al., 2021); uncontrolled blood pressure (systolic≥180 mmHg or diastolic≥110 mmHg) or fibrosis in other organs (Yuan et al., 2014); pregnancy or breastfeeding (Chen et al., 2021); psychiatric or infectious disease.

### Treatment

Qualified patients were randomized in a 1:1 ratio to receive either QSG or a matching placebo for 12 weeks, twice a day, 6.8 g granules at a time. Patients in both groups received internal medicine treatment according to the 2014 guidelines (Zhang and Zhang, 2014). Participants were prohibited from taking other Chinese medicines during the trial period. Beijing KangRenTang Pharmaceutical Co., Ltd. manufactured the QSG and placebo.

### Outcomes and clinical visit

The primary outcome is the proportion of patients demonstrating a more than 30% (4) decrease in NT-proBNP levels after 12 weeks of treatment. The secondary outcomes are changes in NYHA functional degree, 6-min walking distance, echocardiogram data, quality of life measured with the Minnesota Living with Heart Failure Questionnaire (MLHFQ, Table MLHFQ, ©1986 Regents of the University of Minnesota), and symptoms with TCM syndrome integral scale (SIS). We collected their NYHA functional degree, 6 MWD, MLHFQ, and TCM syndrome integral scale information at each visit point (week 0 ± 3 days, week 4 ± 3 days, week 8 ± 3 days, and week 12 ± 3 days). We measured the NT-proBNP and echocardiogram data only at the first and last visits.

TCM SIS is determined based on preliminary clinical research results and expert opinion (Table scale). All laboratory tests were carried out at local participating units. Plasma NT-proBNP was tested using electrochemiluminescence (Roche Diagnostics, Basel, Switzerland).

Any adverse medical event that occurs in a subject during the observation period of a clinical study, regardless of whether it is causally related to the test drug, is considered an adverse event (AE). Serious adverse event (SAE) includes death, life-threatening hospitalization, prolonged hospitalization, or causing a permanent disability, cancer, congenital disabilities, and drug overdose.

### Randomization

An independent statistician who was unaware of the design and purpose of the study generated the randomization table using the Statistical Analysis System (SAS, version 9.4). The 200 eligible patients were randomly allocated in a 1:1 ratio to either the QSG treatment group or the placebo group. The randomization was stratified by the study site. The statistician was not involved in patient recruitment. The random assignment form was sealed in an opaque envelope, and the project leader and statistician kept a copy. Participants received the number in the enrollment order and took the corresponding drugs.

### Blinding

Both participants and investigators were blinded to the treatment allocation throughout the study. QSG and placebo were identical in packaging, particle appearance, and taste. The manufacturer labeled the random codes on the packaging. The clinical trial pharmacist at each center provided packaged drugs to the participants according to the randomization number.

### Sample size calculation

The sample size was calculated based on the primary outcome. According to a previous trial on treating CFH with TCM (4), we assumed that the proportion of patients demonstrating a more than 30% NT-proBNP decrease in the QSG group would be 53% (P2), and the other ratio in the placebo group would be 31.98% (P1). Given a type-I error rate of α = 0.05 and a type-II error rate of β = 0.2, each group required 80 patients. Considering a drop rate of 20% among recruited patients, the total sample size needed 200 to achieve efficacy analysis results.

### Data analysis

Two researchers entered the data independently. We used the per-protocol analysis to analyze the data and the Shapiro-Wilk test to identify data distribution. Normally distributed variables were analyzed by two-tailed t-test between two groups, and paired t-test were used for intragroup data comparisons. Other distribution-type variables were analyzed by the Wilcoxon or Chi-Square test. The MLHFQ and syndrome integral data were analyzed with repeated measures models using time and treatment as the main factors. *p* < 0.05 is a statistically significant criterion.

A subgroup analysis was performed for patients with different levels of LVEF, heart failure with reduced ejection (HFrEF), heart failure with mildly reduced ejection fraction (HFmrEF), and heart failure with preserved ejection fraction (HFpEF). The criteria (Heidenreich et al., 2022) are as follows: HFrEF (LVEF≤40%), HFmrEF (LVEF41%–49%), and HFpEF (LVEF≥50%). First, we compared patients’ baseline age, sex, BMI, and NT-proBNP of patients within each subgroup and confirmed no significant differences. We then compared between-group differences in NT-proBNP and echocardiographic markers in different ejection fraction populations.

All statistical analyses were performed with SAS version 9.4 (SAS Institute Inc., Cary, NC, United States).

## Results

### Demographic and clinical characteristics

**FIGURE 1:**
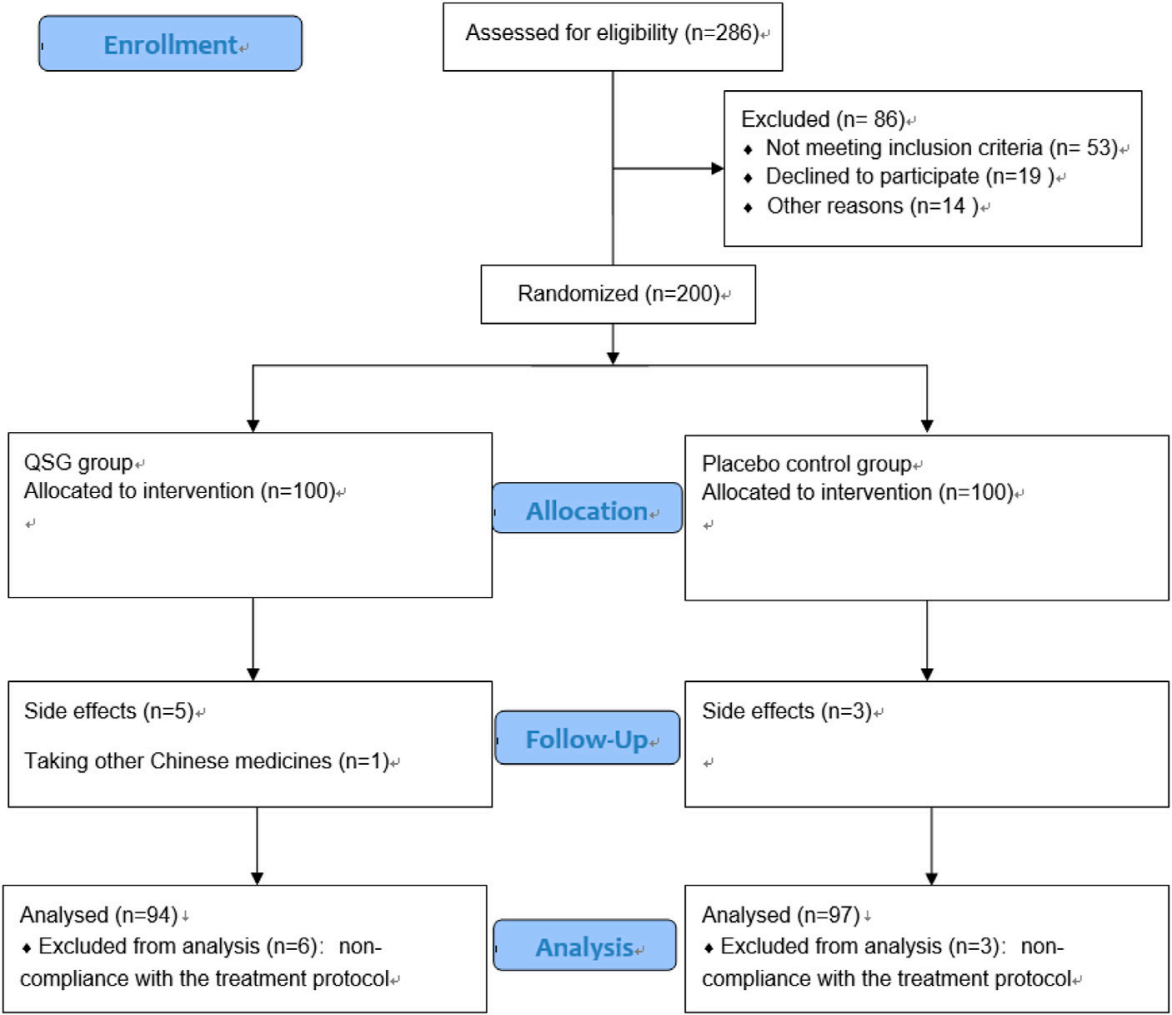
Study flow chart.

From January 2017 to March 2021, 200 patients were recruited to participate in this trial. Nine patients were excluded for non-compliance with the treatment protocol. Finally, there were 94 patients in the QSG group and 97 in the placebo group (Figure 1). The mean age of all patients was 67.95 years, and 62.8% were male. The average course of CHF was 65.32 months. The detailed baseline information is shown in Table 1. No significant difference was found between the two groups at baseline.

**TABLE 1:** Baseline characteristics of patients.

Characteristics	All n = 191	QSG, n = 94	Placebo, n = 97	*p*-value^a^
Male/Female	122/69	59/35	63/34	0.692
Age	70 (63,75)	69 (61,75)	70 (63,75)	0.450
Height (cm)	167 (158,173)	167 (157,173)	166 (158,173)	0.802
Weight (kg)	69.2 ± 11.8	69.0 ± 12.0	69.2 ± 11.5	0.944
BMI	24.9 ± 3.4	24.9 ± 3.6	24.8 ± 3.1	0.775
Heart rate	75 (66,86)	75 (68,85)	76 (65,87)	0.485
Breath	19 (18,20)	19 (18,20)	19 (18,20)	0.575
Sbp	135.6 ± 20.2	136.1 ± 19.2	135.1 ± 21.1	0.389
Dbp	80 (70,88)	80 (70,90)	77 (70,88)	0.115
Mean LVEF	50.5 ± 16.3	51.1 ± 16.2	49.8 ± 16.4	0.591
Course (month)	65.32 ± 65.8	65.5 ± 68.26	65.11 ± 63.70	0.965
**NYHA**				
II	28 (14.7%)	15	13	0.999
III	112 (58.6%)	54	58	0.999
IV	51 (26.7%)	25	26	0.999
**Comorbidity**				
Diabetes	96 (50.3%)	47	49	0.943
Hyperlipidemia	85 (44.5%)	45	40	0.356
Arrhythmia	80 (41.9%)	39	41	0.913
Hypertension	146 (76.4%)	72	74	0.960
Hyperuricemia	15 (7.9%)	9	6	0.384
Valve disease	7 (3.7%)	3	4	0.732
**Medications**				
Statin	122 (63.9%)	63	59	0.373
ACEI/ARB	115 (60.2%)	62	53	0.058
B-blockers	149 (78.0%)	75	74	0.559
Diuretics	132 (69.1%)	56	76	0.260
Ca-blockers	38 (19.9%)	19	19	0.914
Anticoagulants	127 (66.5%)	69	58	0.300
Spironolactone	93 (48.7%)	40	53	0.095
Nitrates	49 (25.7%)	28	21	0.198

Normally distributed data are presented as the mean ± SD, or the percentage of patients. Non-normally distributed data are presented as the median (Q1, Q3). ‘Course’ means chronic heart failure duration.

Sbp = systolic blood pressure; Dbp = diastolic blood pressure; NYHA, new york heart association.

^a^ Based on independent t-test or Wilcoxon test.

### Primary outcome

The baseline NT-proBNP levels in the QSG and placebo groups were comparable. After 12 weeks of treatment, 47 patients (50%) in the QSG group and 31 patients (32%) in the placebo group were defined as effective according to the previous standard (Chi-square 6.431, *p* = 0.011, Table 2). The NT-proBNP numerical value in the two groups demonstrated a significant difference (891.0 (481.8,2443.5) vs. 1,649.0 (659.4,3179.0), pg/ml, *p* = 0.027, Table 3).

**TABLE 2:** Treatment efficiency in NT-proBNP.

	QSG, n = 94	Placebo, n = 97	Chi-square value	*p*-value
Valid (≥30%)	47 (50.0%)	31 (32.0%)	6.431	0.011
Invalid (<30%)	47 (50.0%)	66 (68.0%)		

QSG, Qi shen granules. The effective standard is that the reduction ratio is greater than or equal to 30%.

**TABLE 3:** numerical value change in NT-proBNP.

	QSG, n = 94	Placebo, n = 97	Z	*p*-value
NT-ProBNP baseline	1,633.0 (741.5,3677.7)	1,607.2 (736.0,4682.5)	−0.181	0.857
NT-ProBNP after treat	891.0 (481.8,2443.5)	1,649.0 (659.4,3179.0)	−2.214	0.027

QSG, Qi shen granules. *p* values determined by the Wilcoxon test.

We analyzed the results sorted by LVEF level. First, we compared baseline data, including age, gender BMI, and NT-proBNP, to ensure that the subsequent analysis was reasonable and reliable (Table 4). No significant difference was found in a subset. In the HFrEF subgroup, the treatment efficiency of QSG was better than the control group. At the same time, the rest of the population did not show differences (Table 5).

**TABLE 4:** subgroup baseline characteristics of patients.

Subgroup	Characteristics	t/Z	*p*-value
HFrEF	Age	−0.536	0.562
	Sex	−1.320	0.187
	BMI	0.689	0.494
	NT-proBNP	0.106	0.916
HFmrEF	Age	−1.909	0.056
	Sex	−0.410	0.682
	BMI	−0.616	0.541
	NT-proBNP	−0.828	0.413
HFpEF	Age	−0.534	0.593
	Sex	−0.710	0.478
	BMI	0.346	0.730
	NT-proBNP	0.324	0.746

HFrEF, heart failure with reduced ejection fraction; HFmrEF, heart failure with mildly reduced ejection fraction; HFpEF, heart failure with preserved ejection fraction.

**TABLE 5:** subgroup Treatment efficiency in NT-proBNP.

	Group	Invalid	Valid	*p*-value
HFrEF (56)	QSG	13	14	0.006
	Placebo	24	5	
HFmrEF (38)	QSG	9	3	0.900
	Placebo	19	7	
HFpEF (97)	QSG	25	30	0.364
	Placebo	23	19	

QSG, Qi shen granules; HFrEF, heart failure with reduced ejection fraction; HFmrEF, heart failure with mildly reduced ejection fraction; HFpEF, heart failure with preserved ejection fraction.

The effective standard is that the NT-proBNP, reduction ratio is greater than or equal to 30%.

### Secondary outcome

Echocardiography measurements. In the overall population, echocardiography did not significantly change from baseline to the end. No significant changes were observed between groups (*p* > 0.05 for all, Table 6).

**TABLE 6:** Echocardiographic indices.

		QSG, n = 94	Placebo, n = 97	*p*-value
LVEF	0 W	51 ± 16	50 ± 16	0.597
	12 W	53 ± 14	51 ± 15	0.297
FS	0 W	28 ± 10	26 ± 10	0.382
	12 W	30 ± 11	27 ± 10	0.090
LVEDD	0 W	53 ± 11	55 ± 12	0.222
	12 W	52 ± 12	55 ± 11	0.074
LVESD	0 W	39 ± 12	42 ± 13	0.212
	12 W	39 ± 11	41 ± 13	0.345

QSG, Qi shen granules; LVEDD, left ventricular end-diastolic dimension; LVEF, left ventricle ejection fraction; FS, fractional shortening; LVESD, left ventricular end-systolic dimension.

6MWD. Some participants refused to undergo the 6-min walk test due to physical intolerance or concerns about possible risks. Both groups showed improvements after treatment for 12 weeks (*p* < 0.001 and *p* = 0.050, respectively). Compared to placebo group patients, QSG patients exhibited more significant improvements (Table 7, *p* = 0.001).

**TABLE 7:** 6 MWD.

6 MWD, m	QSG, n = 75	Placebo, n = 84	Z	*p*-value
0 W	330.7 (217.0,384.3)	316.0 (249.2,359.0)	−0.359	0.720
12 W	367.0 (249.2,359.0)	333.5 (282.0,388.0)	−2.854	0.004
Z	−3.604	−2.544		
*p*-value	<0.001	0.011		

**FIGURE 2:**
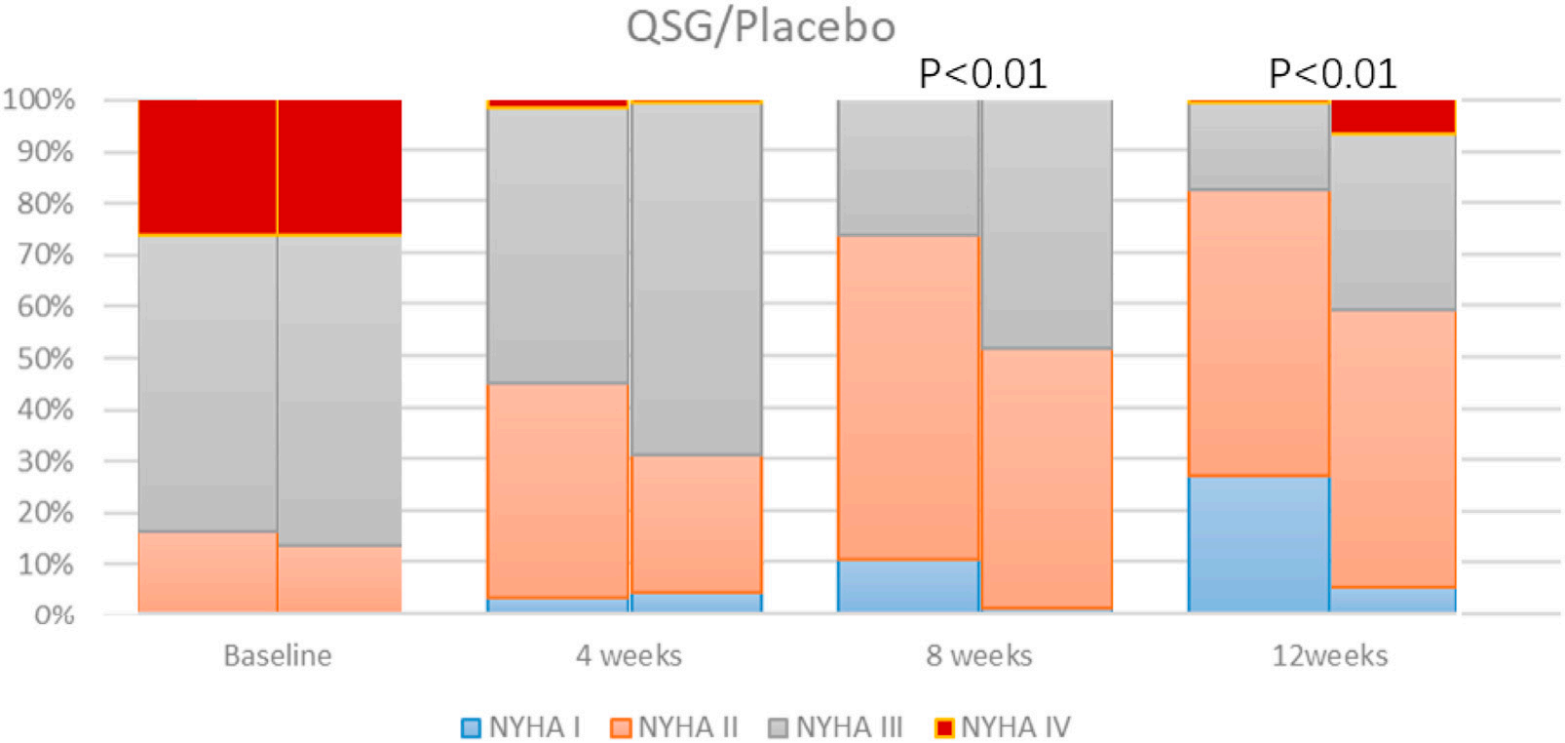
NYHA cardiac function class distribution.

We observed the NYHA class at each visit. There was no significant difference between the two groups at 0 and 4 weeks. As the number of NYHA I-II patients gradually increased, whereas the number of NYHA III-IV patients decreased, the difference between the two groups came up at 8 and 12 weeks (Figure 2). Both patients promoted NYHA function, and the QSG group showed better improvement (*p* < 0.001 at both sites).

**FIGURE 3:**
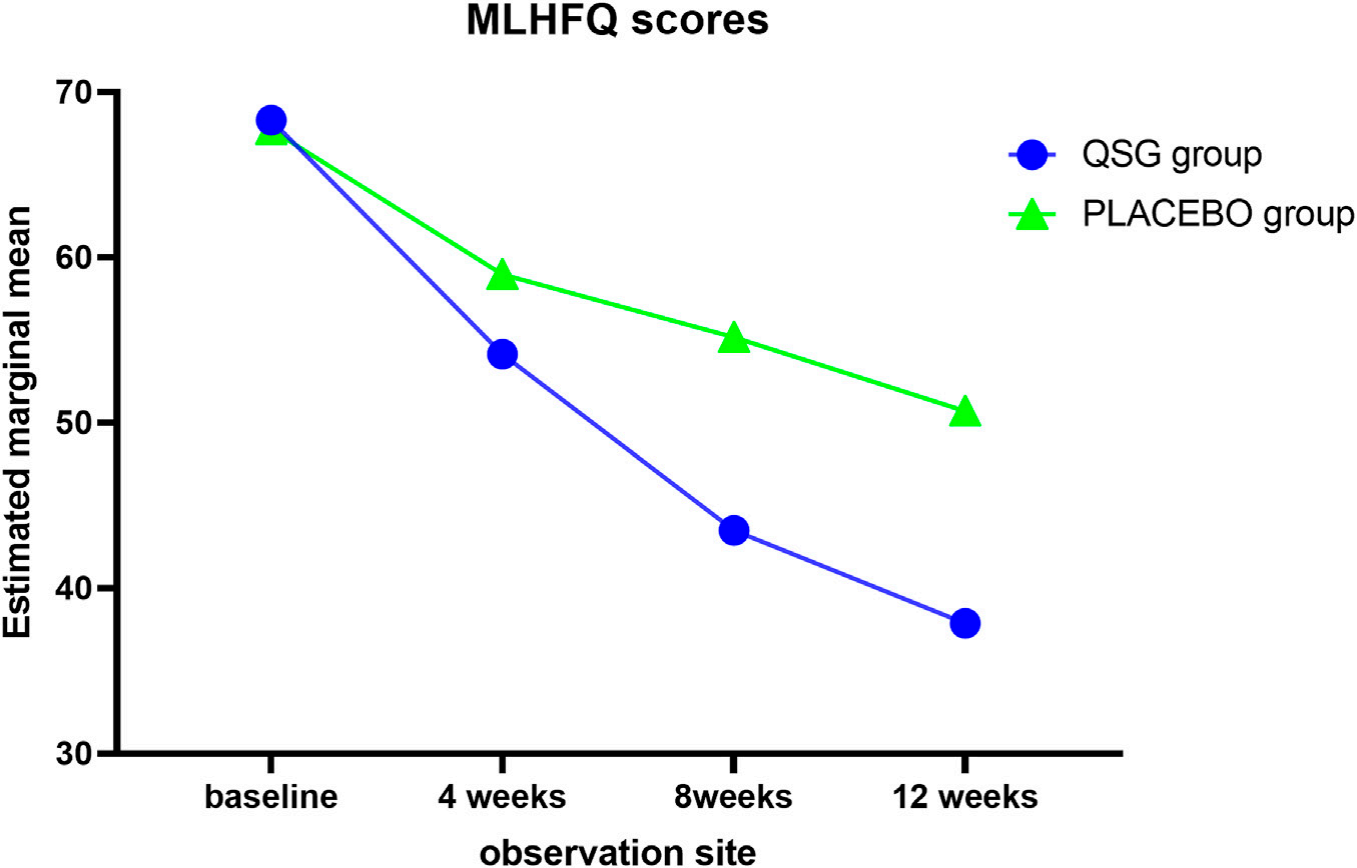
MLHFQ scores.

Quality of life was assessed using the MLHFQ at each visit. The two groups demonstrated a similar mean at 0 weeks and 4 weeks. Significant effects were observed from 8 to 12 weeks (Table 8; Figure 3, *p* < 0.001 for all). In the repeated measures model analysis, patients in the QSG group demonstrated a more significant decrease in the MNSN score (F = 11.669, *p* = 0.001), and the time effect was substantial (F = 98.93, *p* < 0.01).

**TABLE 8:** MLHFQ.

OB (W)	QSG, n = 94	Placebo, n = 97	T Value	*p*-value
0	68.3 ± 14.9	67.7 ± 14.9	0.273	0.785
4	54.1 ± 15.6	59.0 ± 18.7	−1.933	0.055
8	43.5 ± 19.4	55.2 ± 21.2	−3.968	<0.001
12	37.9 ± 21.5	50.7 ± 24.3	−3.860	<0.001

MLHFQ, minnesota living with heart failure Questionnaire; QSG, Qi Shen granules.; OB, observation time.

*p* values were determined by repeated-measures analysis of variance.

**FIGURE 4:**
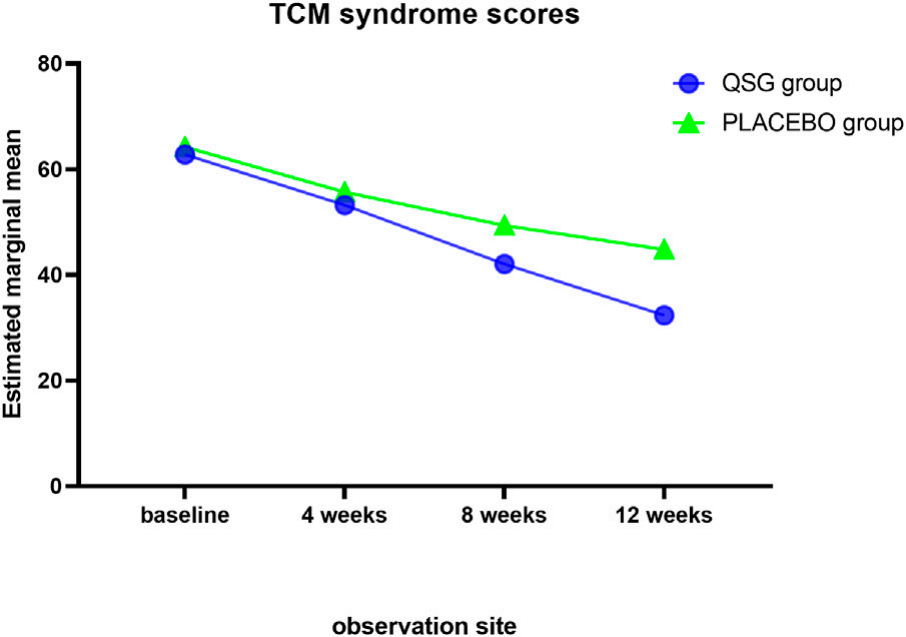
TCM syndrome scores.

The TCM SIS was used to collect syndrome information at each visit. Syndrome scores remained similar in the two groups until 8 weeks. The QSG group exhibited lower scores, meaning that patients alleviated symptoms better than those in the placebo group. (Table 9; Figure 4). In the repeated measures model analysis, significant effects were observed in treatment and time (treatment *p* = 0.0083, time *p* < 0.0001).

**TABLE 9:** TCM syndrome integral scale.

OB (W)	QSG, n = 94	Placebo, n = 97	T Value	*p*-value
0	62.8 ± 14.2	64.2 ± 14.8	−0.686	0.494
4	53.2 ± 18.0	55.7 ± 20.7	−0.885	0.378
8	42.1 ± 19.1	49.4 ± 22.4	−2.412	0.017
12	32.4 ± 23.4	44.8 ± 27.2	−3.380	0.001

QSG, Qi Shen granules.

OB, observation time.

*p* values were determined by repeated-measures analysis of variance.

### Adverse events

The safety set analyses included a total of 191 patients in each group. For adverse events, the total number was five in the QSG group and three in the placebo group. In the QSG group, two patients reported stomachache, two reported nausea, and one reported diarrhea after taking medicine. In the placebo group, two reported nausea, and one reported dry cough. The analysis of drug-induced adverse events revealed no difference between the study groups (Table 10). No serious adverse events (SAEs) related to the study drugs were reported.

**TABLE 10:** Adverse event.

Adverse event	QSG, n = 94	Placebo, n = 97	Chi-square	*p*-value
Yes	5	3	0.590	0.443
No	89	94		

QSG, Qi Shen granules;QSG, for heart failure.

## Discussion

This is the first clinical study demonstrating the QSG effect in heart failure patients. There were changes in the study implementation compared to the previously published protocol: 1) the inclusion criteria added patients with class IV heart function, 2) all patients with TCM diagnosis were included, and 3) a more authoritative and reliable sample size calculation formula was used. We expected to obtain comprehensive and objective information on QSG for heart failure, so the study population was appropriately expanded so that the study results were closer to clinical reality.

The main findings of this study are that QSG could significantly reduce the NT-proBNP level of patients, improve quality of life and the 6-min walk test scores, and alleviate the symptoms of patients. There were no severe adverse reactions during this study. Our observations suggest that QSG could be a supplementary therapeutic strategy for treating heart failure patients.

As mentioned before, the clinical innovation of TCM treatment of heart failure has essential theoretical and practical significance. Currently, TCM treatment of heart failure is limited to tonifying qi, warming yang, and promoting blood circulation. We believe that boosting the effectiveness of TCM treatment of heart failure requires innovation in the therapy. In recent years, the role of heat toxicity in heart failure has attracted attention, partly because the corresponding concept of inflammatory pathways has been valued in the mechanism of heart failure. Based on this theoretical innovation, heat-clearing and detoxifying treatment methods were added to tonifying qi, warming yang, and promoting blood circulation. Due to the lack of comparison with classical treatment methods, the experiment still cannot explain whether the treatment of heat-clearing and detoxification has advantages. However, this research still achieves innovations in therapy.

The role of QSG in treating heart failure has been demonstrated in several animal experiments. Qiu Qi et al. (Qiu et al., 2014) studied the effects on miniature pig models and found that Qishen granules can significantly alleviate the symptoms. QSG have the same effect on heart failure mice and rat models (Xuanchao et al., 2015). Further studies have confirmed that Qishen Granules can inhibit the apoptosis of cardiomyocytes (Chang et al., 2018), the oxidative stress effect induced by p47phox and RAC1, and myocardial fibrosis (Zeng et al., 2018). The regulation mechanism is diverse and resultful. These solid research foundations provide a basis for the clinical use of Qishen Granules.

NT-proBNP is an internationally recognized indicator for the assessment of heart failure. It is effective for both acute and chronic heart failure (Najbjerg et al., 2019). Compared with BNP, NT-proBNP has a longer half-life and a more stable state. It is less affected by renal function indicators (Farnsworth et al., 2018), so NT-proBNP is generally used to evaluate heart failure in clinical practice. As an indicator, a 30% decrease in NT-proBNP is defined as an adequate criterion (Li et al., 2013). This experiment follows this research line of thought, and the results show that QSG can effectively reduce the level of NT-proBNP in patients with chronic heart failure. QSG showed promising results in lowering NT-proBNP in the HFrEF population. However, no significant differences were seen in other patients, suggesting that QSG may be more suitable for application in patients with reduced ejection fraction.

Echocardiography can provide real-time heart function evaluation, tube diameter, ventricular wall and septal thickness, and fractional shortening. It is the preferred method for evaluating cardiac structure and function (Expert Consensus on Standardized Echocardiography, 2019). After treatment, although there was no statistically significant difference in left ventricular ejection fraction between groups, the LVEF value increased, which may be related to the small sample size. There are no apparent differences in many ultrasound indicators, indicating that the changes in the ventricular structure are slowly developing and relatively stable. A significantly different effect on this process may require a longer medication cycle, suggesting the importance of early intervention for heart failure.

Heart function classification can reflect the severity of heart failure as a whole. The results show that the distribution of heart function between groups at baseline is comparable. There was a statistically significant difference between groups at 8 weeks, and the distinction still existed at 12 weeks. This demonstrates that QSG can improve heart function.

Heart failure is a complex and severe disease, and TCM is good at reducing the symptoms of the disease. In this study, QSG reduced heart failure symptoms, enhanced exercise capacity, and improved patients’ quality of life. These metrics may not be solid evidence of efficacy, but patients did benefit from improvements in these metrics. In recent years these soft indicators have played a more critical role in the evaluation of the effectiveness of heart failure (Freedland et al., 2021; Johansson et al., 2021). The main adverse reactions are gastrointestinal types. It is related to the application of aspirin, clopidogrel, and digoxin. The appearance of dry cough may be related to the use of hydrochlorothiazide. There was no difference in adverse reactions between the treatment and control groups, indicating that Qishen granules have good safety.

### Study limitations

Patients with multiple types of ejection fractions were included in this study, and the study population was highly heterogeneous. Unfortunately, there was a lack of positive drug control. Only a comparison with placebo indicated that QSG granules effectively treated chronic heart failure, making it difficult to assess the drug’s actual value in treating heart failure. In future studies, we will optimize the composition and dosage of QSG granules, using qiliqiangxin capsules as a controlled drug, and evaluate the efficacy of QSG granules in treating HFrEF.

## Conclusion

Based on conventional treatment, the randomized, placebo-controlled trial demonstrates the benefits of QSG on the levels of NT-proBNP, symptoms, heart function, 6 MWD, and quality of life for all heart failure patients. Our data suggest that QSG could be used in combination therapy for CHF.

## Data Availability

The raw data supporting the conclusion of this article will be made available by the authors, without undue reservation.

## Abbreviations

CHF, chronic heart failure; FS, fractional shortening; HFrEF, heart failure with reduced ejection fraction; HFmrEF, heart failure with mild reduced ejection fraction; HFpEF, heart failure with preserved ejection fraction; LVEDD, left ventricular end-diastolic dimension; LVEF, left ventricle ejection fraction; LVESD, left ventricular end-systolic dimension; MLHFQ, minnesota living with heart failure questionnaire; NT-proBNP, N-terminal pro-B-type natriuretic peptide; NYHA, new york heart association; QSG, Qi Shen granules; 6 MWD, 6-min walk distance.
